# Mapping the rehabilitation journey of patients with traumatic brain injury in China: a qualitative study

**DOI:** 10.3389/fneur.2026.1768207

**Published:** 2026-03-31

**Authors:** Yijia Li, Mingyang Song, Xuan Zhou, Lanshu Zhou

**Affiliations:** 1 Department of Nursing Clinical Nursing Teaching and Research, Naval Medical University, Shanghai, China; 2 Key Laboratory of Geriatric Long-term Care (Naval Medical University), Ministry of Education, Shanghai, China; 3 The 960th Hospital of the PLA Joint Logistics Support Force, Jinan, China

**Keywords:** care continuity, patient experience, patient journey mapping, rehabilitation nursing, traumatic brain injury

## Abstract

**Background:**

Traumatic brain injury (TBI) rehabilitation involves a complex, multi-stage journey across various healthcare settings. However, current research still lacks a comprehensive understanding of this process from the patient’s perspective. A detailed mapping of the patient journey is crucial for designing targeted and effective rehabilitation strategies.

**Design:**

A qualitative interpretative research design was performed and the standards for reporting qualitative research (SRQR) has been used.

**Objective:**

To construct a patient journey map visualizing the rehabilitation care experiences of adult TBI patients, identify key pain points and service gaps, and provide recommendations for service optimization.

**Methods:**

A qualitative interpretative research design was employed, guided by the Patient-Centered Healthcare Accessibility Framework. Using purposive sampling, 26 TBI patients were recruited from five healthcare settings in a major Chinese city between February and July 2024. Data were collected through 78 semi-structured interviews and 156 h of non-participant observation across acute care, rehabilitation, and community settings. Thematic analysis was conducted using Braun and Clarke’s six-phase framework, followed by process mapping and journey visualization.

**Results:**

The TBI rehabilitation journey comprised three distinct phases: Acute Treatment Phase (2–8 weeks), Continuous Rehabilitation Phase (3–18 months), and Rehabilitation Community Integration Phase (ongoing). Nine emotional states were identified throughout the journey, ranging from fear and helplessness in the acute phase to acceptance and hope in the community integration phase. Thirteen core rehabilitation needs were prioritized across phases. Nineteen significant pain points were categorized into system-level (*n* = 6), provider-level (*n* = 7), and patient/family-level barriers (*n* = 6), with service discontinuity affecting 84% of participants.

**Conclusion:**

The TBI rehabilitation journey is characterized by complexity, non-linearity, and significant service fragmentation. Findings highlight the urgent need for integrated, patient-centered care models with enhanced care coordination, standardized pathways, and comprehensive family support systems. The patient journey map provides a framework for systematic service improvement and policy development.

## 1 Introduction

Traumatic brain injury (TBI) represents a leading cause of neurological disability and mortality worldwide, constituting a major public health challenge with profound long-term consequences for patients, families, and healthcare systems (1–3). Global epidemiological data indicate approximately 50 million new TBI cases annually, with an incidence rate of 64–74 per 100,000 population, and projections suggest TBI will become the third leading cause of death and disability globally by 2030 (4, 5). In China, rapid urbanization and motorization have intensified the TBI burden, with estimated incidence rates ranging from 55.8 to 229.5 per 100,000 population annually, demonstrating significant regional variations and traffic accidents accounting for 60–70% of cases (6–8).

The rehabilitation journey following TBI is inherently complex, typically spanning months to years and involving multiple healthcare settings, disciplines, and care transitions (9, 10). The heterogenous nature of TBI, ranging from mild concussion to severe injury with prolonged coma, necessitates individualized, multiphase rehabilitation approaches addressing physical, cognitive, psychological, and social domains (2, 11, 12). While conventional GCS-based severity classification (mild, moderate, severe) remains in widespread clinical use, there is a growing movement toward more multidimensional frameworks. The recently proposed CBI-M nomenclature—integrating Clinical, Biomarker, Imaging, and Modifier dimensions—reflects an emerging consensus that TBI classification should capture injury complexity and individual modifiers to better guide personalized care (13). Although our study employs conventional severity categories consistent with Chinese clinical practice guidelines (14), this evolving framework underscores the individualization imperative that our findings seek to address. Effective rehabilitation requires coordinated care across acute hospitals, specialized rehabilitation facilities, outpatient services, and community-based programs (15, 16). However, accumulating evidence indicates substantial gaps in service continuity, with patients frequently encountering fragmented care pathways, inadequate information exchange, and insufficient support during critical transition periods (17–23). Qualitative studies consistently demonstrate that discharge and care transitions following acquired brain injury are often experienced as abrupt and poorly coordinated. Patients and families commonly report inadequate preparation for discharge, fragmented or inconsistent information transfer between providers, and the sudden withdrawal of professional guidance upon returning to the community (19, 20).

Despite substantial advances in acute TBI management and rehabilitation interventions (11, 24), understanding of the patient’s lived experience throughout the rehabilitation journey remains limited. Most existing research has focused on clinical outcomes and specific intervention efficacy rather than comprehensively mapping the patient experience across the continuum of care (25–27). Furthermore, when patient experience has been examined, studies have tended to focus on the acute and subacute phases, with the chronic and community integration stages receiving comparatively little attention—despite evidence that patient dissatisfaction and unmet needs are most pronounced precisely in these later phases (28, 29). Patient journey mapping has emerged as a valuable methodology for understanding complex care experiences, identifying service gaps, and informing patient-centered service redesign (30–34). This approach has been successfully applied in various healthcare contexts (35, 36) but remains underutilized in TBI rehabilitation research, particularly in the Chinese healthcare context.

The Chinese healthcare system presents unique characteristics that may influence TBI rehabilitation experiences, including a three-tiered hospital structure, variable insurance coverage, significant out-of-pocket expenses, and heavy reliance on family caregiving (37–39). Understanding how these contextual factors shape the rehabilitation journey is essential for developing culturally appropriate and contextually relevant interventions. Furthermore, the Patient-Centered Healthcare Accessibility Framework (40) provides a comprehensive lens for examining how patients navigate healthcare systems, encompassing dimensions of approachability, acceptability, availability, affordability, and appropriateness of care.

This study aimed to construct a comprehensive patient journey map for adult TBI rehabilitation services, systematically explore patient and caregiver experiences across the rehabilitation continuum, identify critical pain points and service gaps, and develop evidence-based optimization strategies for improving rehabilitation care quality and outcomes.

*Research Question*: What Are the Experiences of the rehabilitation care for people living with Traumatic Brain Injury?

## 2 Methods

### 2.1 Study design

This qualitative study employed an interpretative research design using patient journey mapping methodology (31–34) to explore the rehabilitation care experiences of TBI patients. Patient journey mapping, as both a qualitative research methodology and design thinking activity, enables visualization of patient experiences across healthcare systems and identification of service improvement opportunities. Given that patient journey mapping application remains in early developmental stages with evolving methodological approaches, this study integrated established qualitative methods (non-participant observation and semi-structured interviews) to ensure rigorous data collection.

### 2.2 Research team composition

A multidisciplinary research team was established comprising 9 members: 1 graduate supervisor, 2 master’s students, 1 doctoral student, 3 healthcare professionals specializing in TBI rehabilitation services, and 2 patient representatives. All team members completed standardized training on patient journey mapping methodology. Data collection responsibilities were allocated as follows: non-participant observations were conducted by YL and MS; semi-structured interviews were conducted by YL, MS, and XZ, under the overall supervision of LZ. Through iterative team discussions, we clarified that the journey map’s objective was to comprehensively and visually represent the complete TBI patient rehabilitation experience. Desktop research was conducted to determine journey map components, establishing the basic framework.

### 2.3 Study setting

The study was conducted between February and July 2024 in a major Chinese metropolitan city. Five healthcare institutions representing the complete TBI rehabilitation continuum were purposively selected to capture typical patient pathways within China’s tiered healthcare system: a tertiary hospital neurosurgical ICU (20 beds, ~135 annual TBI admissions), tertiary hospital neurosurgical ward (70 beds, ~433 TBI patients), tertiary hospital rehabilitation department (160 beds, ~296 TBI patients), secondary rehabilitation hospital (60 beds, ~64 TBI patients), and community health service center (~15 annual TBI patients). These settings encompassed acute, subacute, and community-based care phases, reflecting the multi-institutional nature of TBI rehabilitation pathways in China’s healthcare system.

### 2.4 Participants and sampling

#### 2.4.1 Patient participants

Participants were recruited using combined convenience and purposive sampling strategies to ensure maximum variation in rehabilitation experiences. Sampling continued until data saturation was achieved, defined as three consecutive interviews yielding no new themes or insights.

*Inclusion criteria*: (1) Adults (≥18 years) with confirmed TBI diagnosis according to the 2007 Chinese Medical Association Clinical Practice Guidelines for Trauma (34); (2) Patients who had experienced at least one rehabilitation service phase; (3) Patients with stable vital signs and medical condition suitable for study participation; (4) Adequate cognitive capacity for meaningful interview participation or availability of primary caregiver as proxy; (5) Voluntary informed consent and willingness to share detailed rehabilitation journey experiences.

*Exclusion criteria*: (1) Severe cognitive impairment preventing meaningful interview participation without available caregiver proxy; (2) Concurrent serious medical conditions (malignancy, severe infection, significant cardiac/hepatic/renal dysfunction) that could affect rehabilitation trajectory.

*Withdrawal criteria*: (1) Development of acute medical complications during the study period; (2) Voluntary withdrawal from study participation.

#### 2.4.2 Healthcare provider participants

Healthcare providers were recruited from all five study institutions using a combination of convenience and purposive sampling strategies to ensure adequate representation across different professional roles and experience levels. The multidisciplinary participant pool comprised 18 healthcare providers, including neurosurgeons and neurologists (*n* = 4), rehabilitation physicians (*n* = 2), registered nurses from ICU, neurosurgical wards, and rehabilitation units (*n* = 6), physical therapists (*n* = 2), occupational therapists (*n* = 2), speech-language pathologists (*n* = 1), and social workers involved in discharge planning (*n* = 1). Participants had a mean clinical experience of 8.4 years (range 2–22 years), with the distribution including 33.3% with less than 5 years of experience, 38.9% with 5–10 years, and 27.8% with more than 10 years of experience. This diverse sample effectively represented the multidisciplinary teams typically involved in TBI rehabilitation care delivery across the healthcare continuum, capturing perspectives from various stages of patient care from acute intervention through rehabilitation and discharge planning.

### 2.5 Data collection

#### 2.5.1 Preparation phase

Prior to data collection, researchers completed training in non-participant observation and interview techniques. Trust relationships were established with patients, who agreed to notify researchers when transitioning between care settings. Researchers entered observation sites as “trainee nurses” after obtaining informed consent from all relevant parties.

This role was selected as it is a common and socially acceptable presence in clinical settings in China, which facilitated naturalistic observation of routine care processes with minimal disruption. To mitigate the potential Hawthorne effect (where participants alter their behavior due to awareness of being observed), researchers maintained a discreet presence, avoided interference with clinical activities, and conducted prolonged engagement to allow participants to become accustomed to their presence.

#### 2.5.2 Baseline data collection

Demographic and clinical information was collected via structured forms completed by patients or primary caregivers, including: name, gender, age, education level, marital status, insurance type, initial TBI diagnosis date, injury location, injury severity (Glasgow Coma Scale score), and primary caregiver details. Data were verified and updated at each observation/interview session.

#### 2.5.3 Non-participant observation

Structured non-participant observations were conducted by YL and MS across all five study institutions to complement interview data and provide contextual understanding of service delivery processes. Observation protocols focused on patient-provider interaction patterns, care coordination processes, physical environment characteristics and resource availability, workflow efficiency and potential service bottlenecks, and patient and family emotional responses during service encounters. Each observation session lasted 2–4 h, covering different work shifts to ensure comprehensive data collection. Structured observation guides were employed to maintain consistency across settings, with detailed field notes recorded immediately following each session. Observations continued until no new insights emerged, totaling 156 h and generating approximately 30,000 words of field notes documenting key interaction episodes and service delivery patterns across the rehabilitation continuum.

#### 2.5.4 Semi-structured interviews

Interview guides were developed based on literature review and the Patient-Centered Healthcare Accessibility Framework (40). Interviews were conducted by YL, MS, and XZ, under the supervision of LZ. Interviews were audio-recorded with consent, lasting 40–120 min (mean: 75 min for patients/caregivers, 52 min for providers). Each patient/caregiver was interviewed approximately 3 times throughout their journey (see Table 1 for the interview guide), totaling 78 interviews generating >200,000 words of transcribed data. Transcription was completed within 24 h, with member checking for accuracy.

**Table 1:** Interview guide developed based on the patient-centered healthcare accessibility conceptual framework.

Dimension	Key concept	Interview questions
1. Accessibility	*Physical and geographical access to rehabilitation services*	Could you describe in detail the process of transferring to your current rehabilitation facility? What were the reasons for choosing this particular rehabilitation facility? What factors influenced your decision to transfer here?
2. Acceptability	*Patient satisfaction and cultural appropriateness of services*	How do you feel about being transferred to this current rehabilitation facility? What are your overall impressions of this facility? How comfortable do you feel receiving care in this environment?
3. Availability	*Range and quality of rehabilitation services provided*	What specific rehabilitation care services have you received in this current environment? Compared to your previous rehabilitation facility, what differences do you notice in the rehabilitation care services? How would you describe the quality and scope of services available here?
4. Affordability	*Financial accessibility and payment methods*	What difficulties have you encountered during your rehabilitation process, and how were they resolved? What payment method are you using during your rehabilitation period? How manageable are the costs associated with your rehabilitation care?
5. Adaptability	*Responsiveness to individual patient needs and preferences*	What are your current rehabilitation care needs at this stage? To what extent does the current rehabilitation facility meet your current rehabilitation care needs? What gaps exist between the rehabilitation care provided by the current facility and your actual needs? What suggestions do you have for how the current rehabilitation facility could better serve your rehabilitation care needs?

### 2.6 Data analysis

Data analysis followed a systematic multi-stage process:

*Phase 1—thematic analysis*: We employed an abductive analytical approach (41), combining inductive thematic analysis with deductive application of the Patient-Centered Healthcare Accessibility Framework (40). Themes emerged from the data iteratively and were subsequently organized within the theoretical framework to facilitate systematic identification of service gaps.”

*Stage 1—thematic analysis*: Interview transcripts and field notes were analyzed using Braun and Clarke’s six-phase framework (42). In Phase 1 (Familiarization), two researchers (YL, MS) independently read and re-read all transcripts and field notes to achieve deep immersion in the data. Phase 2 (Initial Coding) involved systematic generation of initial codes, facilitated by NVivo 12 software. In Phases 3 and 4, codes were grouped into candidate themes and reviewed iteratively against the full dataset. Phases 5 and 6 involved theme definition, naming, and write-up, with all decisions documented in an audit trail and finalized through team consensus.

Researcher positionality was explicitly addressed prior to and throughout analysis. The senior author (LZ), a qualitative methodology expert, convened regular debriefing sessions in which team members discussed how their professional positions might shape their readings of the data. Reflexivity was maintained through researcher journaling, in which all team members recorded evolving interpretations, emotional responses to participant accounts, and moments when participant experiences challenged prior assumptions. These journals were reviewed collectively during peer debriefing, forming a core component of the audit trail and ensuring that findings authentically represent participants’ perspectives.

*Stage 2—process mapping*: Based on thematic findings, we mapped the rehabilitation journey as a series of components integrating phases and transitions, key activities and touchpoints, emotional trajectories, rehabilitation needs, and pain points and barriers. These thematic findings are presented in detail in the Results section.

*Stage 3—journey visualization*: A comprehensive patient journey map was constructed, integrating phases, activities, emotions, needs, and pain points. The map was iteratively refined through member checking with five participants and expert review by the multidisciplinary research team. It is important to note that the three rehabilitation phases (Acute Treatment, Continuous Rehabilitation, and Community Integration) emerged inductively from the thematic analysis of participant data, rather than being defined *a priori* as part of the analytical framework. These phases reflect the temporal structure of participants’ reported experiences and were subsequently validated through member checking.

### 2.7 Rigor and trustworthiness

Multiple strategies ensured research rigor: (1) prolonged engagement across diverse settings; (2) triangulation of interviews, observations, and documents; (3) member checking with participants; (4) peer debriefing within the research team; (5) detailed audit trail; (6) reflexivity through researcher journaling. The study adhered to SRQR guidelines for reporting qualitative research (43).

### 2.8 Ethical considerations

Ethical approval was obtained from the Naval Medical University Research Ethics Committee. All participants provided written informed consent. Confidentiality and anonymity were maintained through de-identification. Participants could withdraw at any time without consequences.

## 3 Results

### 3.1 Participant characteristics

Participant sociodemographic and clinical characteristics are summarized in Table 2. The study cohort comprised 26 individuals with a mean age of 48.4 years (SD = 12.4, range 21–71). The sample was predominantly male (84.6%, *n* = 22). Educational attainment was categorized as low (50.0%, *n* = 13), middle (30.8%, *n* = 8), or high (19.2%, *n* = 5). The majority of participants were married (85%, *n* = 22). Regarding clinical characteristics, the mean time since injury was 9.5 months (SD = 2.7, range 7–18). TBI severity was distributed as follows: mild (7.6%, *n* = 2), moderate (23.1%, *n* = 6), severe (46.2%, *n* = 12), and very severe (23.1%, *n* = 6). Healthcare financing was primarily through self-payment (61.6%, *n* = 16), followed by Urban Employee Medical Insurance (19.2%, *n* = 5), the New Rural Cooperative Medical Scheme (15.4%, *n* = 4), and Urban Resident Medical Insurance (3.8%, *n* = 1). The primary caregivers were most often spouses (61.6%, *n* = 16), with others including children, siblings, parents, or other relatives; one participant had no primary caregiver.

**Table 2:** Demographic and medical characteristics.

Characteristics	% (*N*) or mean (SD)
Sociodemographics	
Age [years]	48.4 (12.4)
Min/Max age	21–71
Gender	
Male	84.6% (22)
Female	15.4% (4)
Education	
High	19.2% (5)
Middle	30.8% (8)
Low	50.0% (13)
Marital status	
Married	84.6% (22)
Single	15.4% (4)
Medical information	
Duration of illness [months]	9.5 (2.7)
Min/Max duration	7–18
Medical payment method	
Self-pay	61.6% (16)
New Rural Cooperative Medical Scheme	15.4% (4)
Urban Employee Medical Insurance	19.2% (5)
Urban Resident Medical Insurance	3.8% (1)
Injury severity	
Mild	7.6% (2)
Moderate	23.1% (6)
Severe	46.2% (12)
Very severe	23.1% (6)
Primary caregiver	
Spouse	61.6% (16)
Child	11.6% (3)
Parent	7.6% (2)
Sibling	11.6% (3)
Other relative	3.8% (1)
None	3.8% (1)

### 3.2 The TBI rehabilitation journey: a visual mapping of three distinct phases

Through thematic analysis of interview transcripts and observational data, we constructed a comprehensive patient journey map that visualizes the TBI rehabilitation pathway across three temporal phases: Acute Treatment Phase, Continuous Rehabilitation Phase, and Rehabilitation Community Integration Phase.

#### 3.2.1 Process map and trajectory analysis

**Figure 1:**
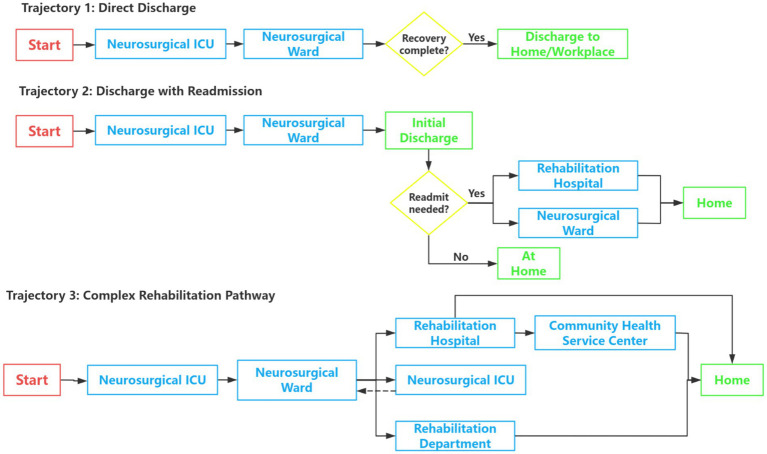
Process map of patients with TBI.

The process map (Figure 1) illustrates the multi-institutional and non-linear nature of the TBI rehabilitation journey. Analysis revealed three predominant trajectories spanning five healthcare settings. A striking 84.6% (*n* = 22) of participants experienced transfers between multiple facilities, with a mean of 3.2 different healthcare settings per patient, while only 15.4% (*n* = 4) followed relatively direct trajectories. The journey typically originated in the neurosurgical ICU, progressed through neurosurgical wards and specialized rehabilitation departments/hospitals, and culminated in a transition to home or community-based care. The map visually underscores the numerous decision points and potential pathways, highlighting significant challenges in care continuity.

It is important to note that the predominance of Neurosurgical ICU admission across all three trajectories reflects the characteristics of the study sample and the Chinese clinical context. The majority of participants sustained severe or very severe TBI (69.2%, *n* = 18), for whom ICU admission represents standard clinical management. Additionally, prevailing clinical norms and medicolegal considerations in China frequently result in ICU admission even for moderate injuries pending neurological monitoring. Consequently, the patient pathway beginning directly from emergency department discharge to home was not captured in the present sample. This constitutes a recognized limitation of our sampling strategy, which required participants to have experienced at least one phase of facility-based rehabilitation. Future research should specifically map the care trajectory of patients with mild TBI managed entirely outside inpatient settings, a population with distinct and potentially underrecognized rehabilitation needs.

#### 3.2.2 Patient journey map

**Figure 2:**
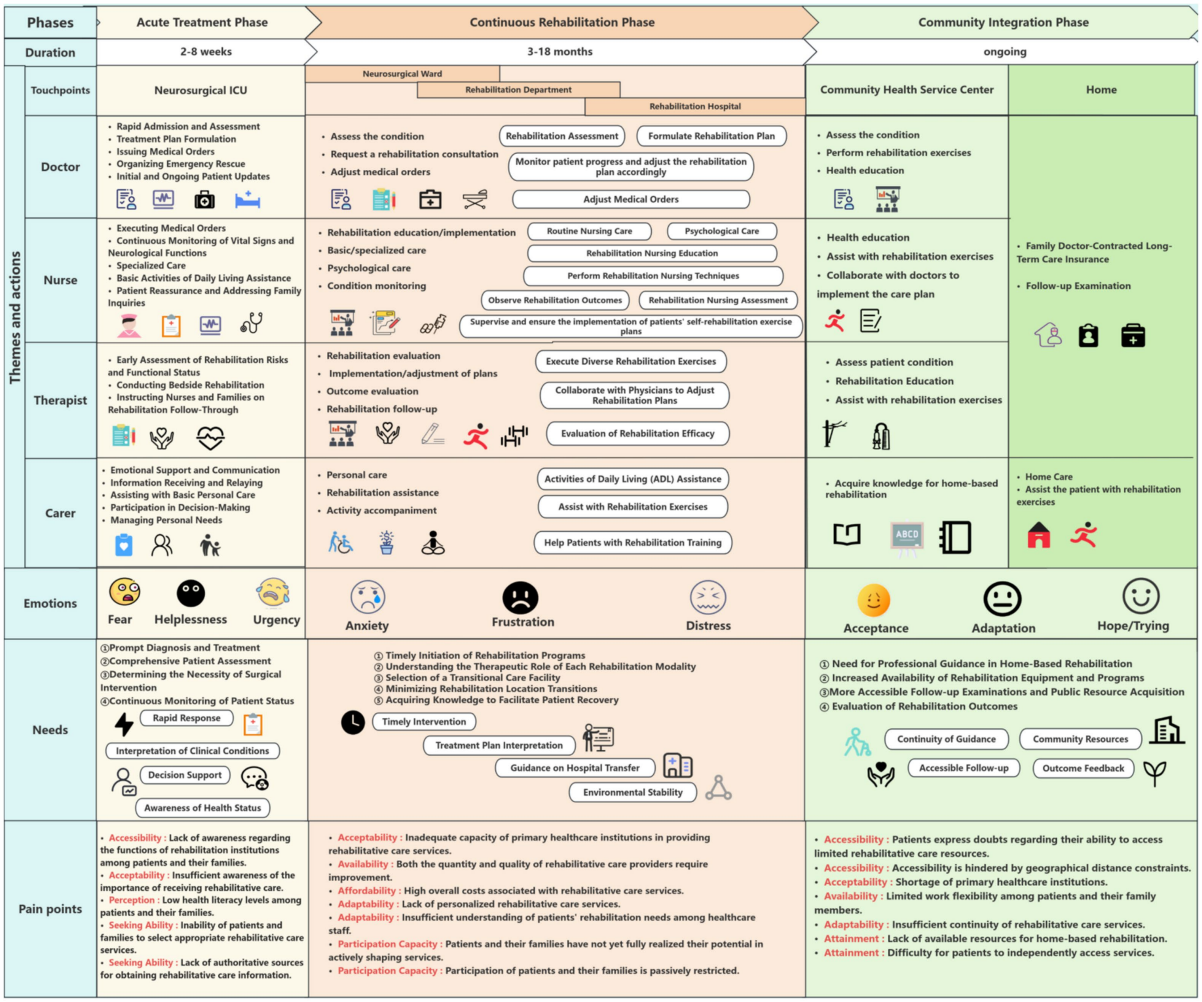
Patient journey map: needs and pain points of patients with TBI. Pain points are identified at critical touchpoints across three phases. For detailed descriptions of each pain point and corresponding mitigation strategies, see Table 3.

The patient journey map (Figure 2) synthesizes the process map with rich qualitative data on tasks, emotions, needs, and pain points across the three phases.

**Table 3:** Comprehensive overview of pain points in TBI rehabilitation care.

Dimension	Corresponding ability	Pain point No.	Pain point description	Data source	Primary phase of occurrence
Approachability	Perception ability	1	*Lack of knowledge about rehabilitation facility functions*: Patients and families are unaware that primary care institutions like community health centers can provide rehabilitation services.	Interviews	Community Integration Phase
		2	*Doubts about accessing limited resources*: Patients are uncertain about their ability to secure resources, such as available beds or insurance eligibility.	Interviews	Continuous Rehabilitation, Community Integration
		3	*Geographical and transportation barriers*: Inconvenient transportation, long distances, and the need for multiple transfers hinder physical access to services.	Interviews	Continuous Rehabilitation, Community Integration
Acceptability	Seeking ability	4	*Insufficient awareness of the importance of rehabilitation*: Rehabilitation is often perceived as an optional supplement rather than an essential component of treatment.	Interviews	Continuous Rehabilitation, Community Integration
		5	*Inadequate service capacity of primary care institutions*: Community health centers lack the specialized capability and resources to provide TBI-specific rehabilitation.	Interviews, Observation	Community Integration Phase
		6	*Lack of primary care institutions*: An absence of local community health centers in the patient’s residential area.	Interviews	Community Integration Phase
Availability & accommodation	Reaching ability	7	*Insufficient quantity and quality of rehabilitation providers*: Shortage of healthcare professionals, heavy workloads, and underutilized role of rehabilitation specialist nurses.	Non-participant Observation	All Phases
		8	*Inflexible work arrangements for patients and families*: Inability to take time off work prevents consistent attendance at rehabilitation sessions or consultations.	Interviews	All Phases
Affordability	Paying ability	9	*High total cost of rehabilitation services*: The cumulative financial burden of long-term care forces many families to discontinue rehabilitation prematurely.	Interviews	All Phases
Adaptability	Engagement ability	10	*Lack of individualized rehabilitation planning*: Limited resources and high workloads prevent providers from tailoring services to individual patient needs.	Non-participant Observation	Continuous Rehabilitation, Community Integration
		11	*Insufficient continuity of care*: Poor coordination and information transfer between different healthcare institutions lead to fragmented rehabilitation.	Non-participant Observation	All Phases
		12	*Providers’ limited understanding of patient rehabilitation needs*: The clinical focus remains on the disease itself, with inadequate attention to proactively identifying and addressing patients’ personalized needs.	Interviews, Observation	All Phases
(Demand-side)	Perception ability	13	*Low health literacy among patients and families*: Lack of knowledge about the disease and rehabilitation leads to unrecognized needs and reluctance to voice concerns.	Interviews	All Phases
(Demand-side)	Seeking ability	14	*Lack of ability to choose rehabilitation services*: Patients and families lack the knowledge and skills to make informed choices about facilities or treatment plans.	Interviews	Continuous Rehabilitation, Community Integration
		15	*Lack of authoritative sources for rehabilitation information*: Information channels are fragmented and of variable quality, with a lack of official, authoritative platforms.	Interviews	Continuous Rehabilitation, Community Integration
(Demand-side)	Reaching ability	16	*Lack of resources for home-based rehabilitation*: Scarcity of rehabilitation equipment and dedicated spaces in home and community environments.	Interviews	Community Integration Phase
		17	*Inability to access services independently*: Physical and cognitive impairments prevent patients from accessing services without assistance.	Interviews	All Phases
(Demand-side)	Engagement ability	18	*Limited active participation in shaping services*: Patients lack the knowledge for shared decision-making, and healthcare institutions do not provide adequate opportunities for involvement.	Non-participant Observation	Continuous Rehabilitation, Community Integration
		19	*Passive and restricted participation of patients and families*: Patients are limited by their condition, and caregivers are constrained by time or capacity, resulting in a predominantly passive role.	Interviews, Observation	Continuous Rehabilitation, Community Integration

##### 3.2.2.1 Phase 1: Acute Treatment Phase (duration: 2–8 weeks; primary setting: neurosurgical ICU)

*Predominant emotions*: Fear, helplessness, and urgency dominated this life-saving stage. Patients reported fragmented memories and a terrifying loss of bodily control. As P23 recounted, “My mind was filled with disjointed pictures and noises; I felt like I was falling into an abyss… The loss of bodily control was profoundly frightening.” Family members experienced intense anxiety and information asymmetry, often feeling powerless in critical decision-making.

*Core needs*: Needs were centered on rapid diagnosis and treatment, comprehending the patient’s condition and prognosis, guidance on surgical decisions, and real-time updates on the patient’s status.

##### 3.2.2.2 Phase 2: Continuous Rehabilitation Phase (duration: 3–18 months; primary settings: neurosurgical wards, rehabilitation departments, specialized hospitals)

*Predominant emotions*: This phase was characterized by anxiety about long-term outcomes, frustration from slow progress and plateaus, and distress over the immense financial and caregiving burdens. P6 expressed the torment of fluctuation: “Some days you feel a slight improvement, only to regress the next. This constant back-and-forth is utterly tormenting.”

*Core needs*: Key needs included the prompt initiation of rehabilitation programs, understanding the purpose of various interventions, making informed choices about facility transfers, minimizing frequent location changes, and acquiring knowledge to aid recovery. P14 articulated the disorientation of facility transfers: “No one told us where to go next or what to expect. We felt completely alone in making this decision.” P8 described the challenge of uninformed choice: “We did not know how to evaluate which rehabilitation hospital was better—we just picked the closest one.”

##### 3.2.2.3 Phase 3: Community Integration Phase (duration: ongoing; primary settings: community health centers, home)

*Predominant emotions*: After a protracted journey, emotional states evolved toward acceptance, adaptation, and a forward-looking hope. Patients like P10 demonstrated resilience: “I understand that some functions might not return. Instead, I focus on what I can do now and how to do it well.”

*Core needs*: Needs shifted toward professional guidance for home-based rehabilitation, access to more rehabilitation resources and public facilities, convenient follow-up services, and methods to evaluate long-term rehabilitation effectiveness. P19 described the abrupt transition to home-based care: “When we finally went home, I thought the hard part was over. But there was no one to guide us on what exercises to continue, and the community clinic staff had never treated a TBI patient before.”

### 3.3 Pain point analysis: a framework of 19 systemic barriers

Guided by the Patient-Centered Healthcare Accessibility Framework (40), we identified 19 distinct pain points hindering optimal rehabilitation care. These were categorized across the framework’s five dimensions of accessibility and five corresponding patient capabilities, as detailed in Table 3.

P11 captured the relational cost of these transitions: “Every time we moved to a new place, it felt like starting over. The new therapists did not know my story, did not understand what we’d been through. I had to prove myself again.”

### 3.4 Member checking and validation

To ensure the trustworthiness and accuracy of the findings, the process map and patient journey map were validated through member-checking discussions with eight participants (four patients/caregivers and four healthcare providers). All participants confirmed the maps’ comprehensive and accurate representation of the rehabilitation experience. Minor refinements were made to the emotional trajectory timing based on their feedback, resulting in final versions that received unanimous endorsement.

## 4 Discussion

This study provides the first comprehensive patient journey map of TBI rehabilitation experiences in China, revealing a complex, non-linear trajectory characterized by significant service fragmentation, emotional volatility, and multilevel barriers. The findings extend beyond descriptive documentation to illuminate fundamental structural and systemic issues that impede optimal rehabilitation outcomes. In this discussion, we critically examine our findings through multiple theoretical lenses, situate them within the broader international literature, explore the contextual specificity of the Chinese healthcare system, and propose evidence-based strategies for service transformation.

### 4.1 Systemic roots of service fragmentation and continuity of care failures

This study found that 84.6% of participants experienced service discontinuity across multiple care transitions, reflecting systemic fragmentation within China’s TBI rehabilitation service system. From the Complex Adaptive Systems (CAS) theory perspective, this fragmentation is not merely isolated coordination failures but rather emerges from the structural and dynamic properties of the healthcare system (44). Acute hospitals in China are primarily reimbursed for medical procedures and inpatient days, incentivizing rapid discharge regardless of patients’ rehabilitation readiness (45). Conversely, rehabilitation facilities face capacity constraints and insurance restrictions that limit admission criteria and length of stay. These structural misalignments generate the “premature discharge” phenomenon reported by 73% of our participants, where clinical transitions are driven by administrative and financial imperatives rather than patient readiness. Second, professional specialization and siloed care delivery fragment the rehabilitation pathway into discrete, poorly connected episodes. Our participants described encountering different professional teams at each transition point with minimal cross-communication or shared care planning. This reflects what CAS theory identifies as weak coupling between system components (46). Beyond clinical and experiential consequences, premature discharge and insufficient transitional support carry substantial economic implications that are critical for policy advocacy. While early discharge may appear to contain acute care costs, evidence from other healthcare systems indicates a ‘cost-shifting’ effect: patients discharged without adequate rehabilitation readiness face elevated risks of complications, emergency readmissions, and long-term functional dependency, all of which generate significant downstream expenditure for healthcare systems and society. Conversely, investment in coordinated transitional care and community rehabilitation has been associated with net cost savings through reduced rehospitalization and improved long-term productivity outcomes, including return to work. In the Chinese context, where families bear an estimated 60–80% of rehabilitation costs (47), the economic burden of service gaps extends well beyond the healthcare system into household financial catastrophe—a dimension that future health economics research in this population should urgently address.

From the Continuity of Care theory perspective, Haggerty et al.’s (48) framework identifies three dimensions of continuity: informational, management, and relational. Our findings demonstrate systematic failures across all three dimensions in the Chinese TBI rehabilitation context. Informational discontinuity was pervasive, with 88% of participants reporting that information did not transfer effectively between settings. Families described carrying paper records, repeating medical histories to each new provider, and encountering contradictory information. This reflects the fragmented health information technology landscape in China, where hospitals operate independent electronic systems with minimal interoperability (49). Management discontinuity manifested as abrupt changes in rehabilitation approaches, conflicting therapeutic recommendations, and lack of longitudinal care planning. Participants described each facility as “starting from scratch,” with new assessments, new goals, and new strategies that bore little relation to prior care. This finding aligns with international research documenting that care transitions in TBI rehabilitation are frequently characterized by inadequate discharge planning, poor communication between sending and receiving providers, and lack of transitional care protocols (50). Piccenna et al. (19), in a qualitative systematic review, found that patients with acquired brain injury consistently described discharge as abrupt and disorienting, with little preparation for community life. Abrahamson et al. (20) similarly reported that patients with severe TBI could not identify any coherent transition plan upon discharge, with community services perceived as short-term and insufficient. That such patterns are documented across the United States, Australia, the United Kingdom, and China suggests that service discontinuity at care transitions is a structural feature of complex rehabilitation systems, not merely a product of any single healthcare model. Relational discontinuity emerged as particularly distressing for participants, who described losing trusted therapeutic relationships at each transition. As illustrated by P11’s account in the results section, the loss of established therapeutic relationships at each transition was not merely emotionally distressing but had functional consequences, undermining rehabilitation engagement and continuity of goals.

### 4.2 Emotional trajectories, psychological processes, and rehabilitation engagement

In rehabilitation contexts, self-efficacy has been shown to predict functional independence, treatment adherence, and psychological adjustment after acquired brain injury (51, 52). Our finding that emotional states shifted from “helplessness” in the acute phase to “determination” and “acceptance” in later phases suggests a trajectory of self-efficacy development. However, this trajectory was repeatedly disrupted by service discontinuities, confusing information, and unmet needs. Acceptance of disability—characterized by acknowledging limitations while maintaining positive self-regard and life engagement—has emerged as a critical mediator of psychological outcomes after TBI (53). Our finding that “acceptance” emerged as a dominant emotional state in the community integration phase aligns with this literature, suggesting that acceptance develops gradually as patients confront persistent limitations and adapt expectations. However, our data also reveal that the pathway to acceptance was profoundly shaped by service system characteristics. Participants who experienced better care continuity, clearer information, and stronger support networks described more adaptive acceptance characterized by hope and resilience. Conversely, those who encountered severe service gaps and overwhelming burdens described acceptance tinged with resignation and despair. These patterns are corroborated by Hoepner and Keegan (28), who found that repeated encounters with providers lacking TBI-specific knowledge progressively eroded patients’ trust in healthcare systems, directly undermining self-efficacy and willingness to engage with rehabilitation. Complementing this, Hoepner et al. (29) demonstrated that patient satisfaction with healthcare providers declined significantly from the acute to the chronic phase, with the sharpest drop occurring at the point of community transition—empirically anchoring our finding that the community integration phase is the most emotionally taxing and least adequately supported stage of the TBI journey.

### 4.3 Contextual specificity: Chinese versus Western TBI rehabilitation systems

A critical contribution of our study is illuminating how the Chinese healthcare context shapes TBI rehabilitation experiences in ways that differ substantially from Western systems. Understanding these contextual differences is essential for interpreting our findings and adapting intervention models developed in other settings. China’s three-tiered hospital system (primary, secondary, tertiary) creates a hierarchical structure where specialized rehabilitation services concentrate in urban tertiary hospitals, with limited capacity at lower tiers (54). This contrasts with many Western systems where rehabilitation services are more distributed across specialized rehabilitation hospitals, outpatient centers, and community programs. The concentration of expertise in tertiary centers creates geographic access barriers for rural and remote populations. Moreover, China’s healthcare financing relies heavily on out-of-pocket payments despite expanding insurance coverage (55). Our finding that 100% of participants experienced overwhelming financial burden reflects this reality. While China’s basic medical insurance schemes have expanded coverage to over 95% of the population, rehabilitation services face significant coverage limitations: arbitrary session limits, exclusion of many therapy types, and low reimbursement rates that leave families bearing 60–80% of rehabilitation costs (47). This contrasts sharply with countries like Australia, Germany, or Scandinavian nations where comprehensive public insurance or social insurance schemes provide extensive rehabilitation coverage with minimal patient cost-sharing (56).

A striking finding was the intensive, sustained family caregiving role in Chinese TBI rehabilitation. All participants relied heavily on family members for physical care, emotional support, financial resources, care navigation, and decision-making. Family members frequently left employment to provide full-time care, with profound economic and personal consequences. This reflects deeply rooted cultural expectations of filial piety and family responsibility in Chinese society (57). While family caregiving is certainly important in Western TBI rehabilitation, the expectation and intensity differ. Western systems more commonly employ professional home care services, respite programs, and support services that complement family caregiving (58). In contrast, China’s formal home care and community support infrastructure remains underdeveloped, leaving families to shoulder near-total caregiving responsibility (59). A fundamental structural difference between Chinese and Western TBI rehabilitation systems is the presence (or absence) of formalized care coordination mechanisms. Many Western systems, particularly in the United States, Canada, and Australia, have established TBI model systems with designated case managers or care coordinators who provide longitudinal support, facilitate transitions, coordinate services, and advocate for patients and families (60). In contrast, no such role exists in the Chinese healthcare system. Our participants described navigating complex transitions entirely on their own, with families serving as defacto care coordinators despite lacking medical knowledge, system familiarity, or institutional authority.

### 4.4 Limitations

This study has several limitations. First, the single-city setting restricts the transferability of findings to rural and remote regions of China, where healthcare infrastructure and access to rehabilitation services differ substantially. Second, the sampling strategy required participants to have experienced at least one phase of facility-based rehabilitation, excluding patients with mild TBI managed entirely in outpatient or community settings—an important but underexplored population whose trajectories warrant dedicated future investigation. Third, the predominantly male sample (84.6%) reflects TBI epidemiology in China but limits gender-specific analysis of the rehabilitation journey. Fourth, the potential for researcher positional bias—given the team’s clinical nursing background—cannot be fully eliminated despite the use of reflexive journals and peer debriefing. Future research should address these gaps through multi-site, rural–urban comparative designs, longitudinal prospective tracking, and intervention studies testing care coordination models and technology-enabled support systems. Health economics research quantifying the downstream costs of service fragmentation would further strengthen the evidence base for policy reform.

## 5 Conclusion

In conclusion, this study employs patient journey mapping to render visible the often “unreal” and fragmented experience of TBI rehabilitation in China. By moving beyond a siloed examination of discrete service points to a holistic view of the patient’s trajectory, we have identified continuity of care as the central nervous system of an effective rehabilitation pathway—a system that is currently compromised. The 19 pain points detailed herein provide a clear and actionable agenda for improvement. Future efforts must focus on systemic integration, leveraging both human-centered roles like navigators and technology-enabled solutions to create a seamless, supportive, and truly patient-centered journey from trauma to community reintegration.

## Data Availability

The original contributions presented in the study are included in the article/supplementary material, further inquiries can be directed to the corresponding author.
